# Use of cervicovaginal PAMG-1 protein as a predictor of delivery within seven days in pregnancies at risk of premature birth

**DOI:** 10.1186/s12884-017-1427-0

**Published:** 2017-07-26

**Authors:** Yasemin Çekmez, Gürkan Kıran, Esra Tuştaş Haberal, Merve Dizdar

**Affiliations:** Department of Obstetrics and Gynaecology, Umraniye Medical and Research Hospital, Istanbul, Turkey

**Keywords:** PAMG-1, Fibronectin, Preterm labour, Prematurity

## Abstract

**Background:**

To investigate the utility of vaginal placental alpha microglobulin-1 (PAMG-1) protein as a predictor of preterm delivery within 7 days in pregnancies at risk of premature birth.

**Methods:**

This prospective study was performed in women at risk of premature birth. The levels of vaginal PAMG-1 and foetal fibronectin (fFN) and the transvaginal cervical length measurement (CLM) were investigated and compared.

**Results:**

Seventy-two pregnant women were included in this study. The sensitivities of PAMG-1, fFN and CLM were 73.3, 73.6%, and 52.9%, respectively, while the specificities of PAMG-1, fFN and CLM were 92.9%, 94.3%, and 90.9%, respectively. The positive predictive values of PAMG-1, fFN and CLM were 73.3%, 82.3%, and 64.2%, respectively, and the negative predictive values of PAMG-1, fFN and CLM were 92.9%, 90.9%, and 86.2%, respectively.

**Conclusion:**

The diagnostic accuracy of PAMG-1 is similar to that of fFN in terms of preterm labour detection within 7 days.

## Background

Preterm birth (PTB), which is defined as the delivery of an infant before completion of 37 weeks gestation, is responsible for a significant percentage of neonatal morbidity and mortality [1]. Accurate identification of women who are truly in preterm labour allows the appropriate application of interventions such as antenatal corticosteroid therapy, prophylaxis for group B streptococcal infection, magnesium sulphate for neuroprotection and transfer of the patient to a facility with an appropriate nursery level, if necessary, which can all improve neonatal outcome. Conversely, accurate triage of women who are not actually in preterm labour can avoid unnecessary interventions and associated costs [2].

Several biomarkers have been tested for their ability to predict the estimated delivery date based on known risk factors and pathways of preterm birth [3]. In particular, fFN and CLM have been shown to have a good negative predictive value in regards to imminent preterm birth [4, 5]. PAMG-1 is a 34-kDa protein found within amniotic fluid throughout pregnancy. Its concentration in amniotic fluid is greater than 1000-fold that found in normal vaginal secretions or maternal blood [6]. Recently, a rapid slide test that uses immunochromatography methods to detect trace amounts of PAMG-1 in cervicovaginal fluid has been developed to detect the rupture of membranes; the sensitivity of this method ranged from 94.4 to 98.9%, while the specificity ranged from 87.5 to 100% [6, 7].

In this study, we investigated the presence of PAMG-1 in the cervicovaginal fluid of women with clinical evidence of preterm labour without membrane rupture. We then raised the question of whether vaginal PAMG-1 can be used as a predictor of preterm labour within 7 days.

## Methods

### Study population

The study population of this prospective study consists of pregnant women with singleton pregnancies between 24 + 0 and 34 + 0 weeks of gestation who were admitted consecutively to the Obstetrical Department of the Umraniye Medical and Research Hospital, İstanbul, Turkey, for preterm birth risk between Sep 2015 and Sep 2016.

Upon initial evaluation, a detailed history and a sterile speculum exam followed by a digital exam, cervical length measurement with transvaginal ultrasound, obstetrical ultrasound and external cardiotocography were performed in all subjects. Women with all of the following clinically evaluated symptoms of preterm labour were eligible for inclusion in the study: at least four contractions in 60 min based on external cardiotocography, cervical dilation of >1 cm to <3 cm, effacement of >50%, and a cervical length of <30 mm on transvaginal ultrasound. This study was approved by the Ethics Committee of Umraniye Medical and Research Hospital. Written informed consent was obtained from each participant. Women with ruptured membranes that were evident either by the pooling of fluid or that were detected by the Amnisure® test (for women who demonstrated no pooling), active bleeding, multiple pregnancies, growth restriction, foetal anomalies, placental anomalies, history of coitus within 24 h, history of preterm birth, preeclampsia or signs of any infection were excluded from the study.

### Sample collection and test procedures

**Fig. 1:**
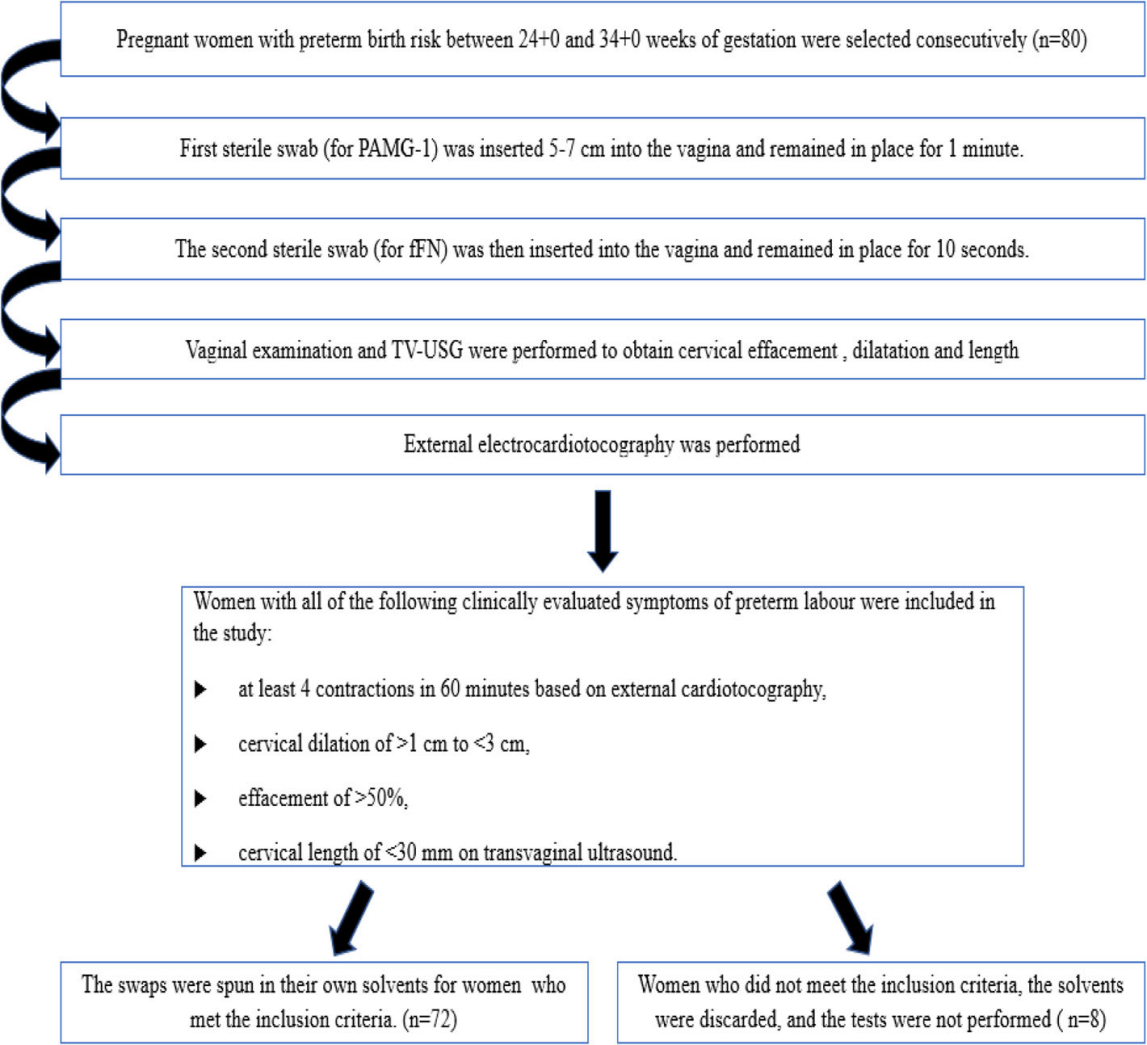
Flow chart of sample collection and test procedures

The PAMG-1 and fFN tests were performed according to the manufacturer’s instructions. At the time of the initial evaluation, the first sterile swab (for PAMG-1) was inserted 5–7 cm into the vagina and remained in place for 1 min. The second sterile swab (for fFN) was then inserted into the vagina and remained in place for 10 s before the vaginal examination. After the swabs were removed, a vaginal examination, transvaginal ultrasound and external electrocardiotocography were performed, and if the women met the inclusion criteria, the swabs were spun in their own solvents (Fig. 1). When the women did not meet the inclusion criteria, the solvents were discarded, and the tests were not performed. The appearance of two lines on the strips was indicative of a positive result for both tests, as stated in the manufacturer’s instructions. All tests were interpreted and collected by the same two investigators.

### Follow-up procedure

Decisions regarding admission, antenatal corticosteroid therapy, prophylaxis for group B streptococcal infection, and magnesium sulphate for neuroprotection were performed according to the Committee on Practice Bulletins-Obstetrics no. 159 of the American College of Obstetricians and Gynecologists [8]. All the participants were followed-up until their delivery. The gestational week at delivery and the type of labour were recorded in order to assign an interval between the test and the delivery. The primary outcome was delivery within 7 days.

### Statistical analyses

Sensitivities (SNs), specificities (SPs), positive predictive values (PPVs), and negative predictive values (NPVs) were calculated for the detection of PAMG-1, fFN and CLM and were compared with each other using McNemar’s exact test; 95% confidence intervals were calculated using the Clopper-Pearson method. A value of *p* < 0.05 was considered statistically significant.

## Results

A total of 72 pregnant women were included in this prospective study after eight were excluded due to the absence of obligatory selection criteria. Four of the eight excluded patients had less than four contractions in 60 min on external electrocardiotocography, three of them had more than 3 cm cervical dilatation and one of them had more than 50% cervical effacement. PAMG-1, CLM and fFN were detected in all participants. The characteristics of the patients are listed in Table 1. The mean cervical length was 20.4 mm ± 3.8 at admission.

**Table 1:** Patient characteristics

Characteristics of patients	
Age (Mean ± SD) years	26 ± 2.6
Gravidity (Mean ± SD)	1.15 ± 0.9
Parity (Mean ± SD)	0.9 ± 0.2
Mean number of gestational weeks at diagnosis of threatened preterm birth (Mean ± SD)	32.4 ± 1.8
Mean number of gestational weeks at delivery (Mean ± SD)	34.3 ± 1.1
Percentage of women who delivered within 7 days (Mean ± SD)	20.8%

Among this delivery group, women in whom PAMG-1 and fFN were determined to be positive were significantly more likely to deliver within 7 days than those who were negative for PAMG-1 and fFN. Eleven of 15 women who were positive for PAMG-1, 4 of 57 who were negative for PAMG-1, 14 of 19 who were positive for fFN, and three of 53 who were negative for fFN delivered within 7 days (11/15 vs 4/57 OR: 6.4 95%CI 1–24.2), (14/19 vs 3/53 OR: 5.4 95%CI 1.4–34.6) (CI: confidence interval, OR: odds ratio).

Delivery within 7 days occurred in 15/72 (20.8%) of the pregnant women (Table 1). Seven of these women were below 34 weeks of gestation, while the remaining eight were below 37 weeks of gestation. This rate was found to be 73.3% for patients with positive PAMG-1 test results, 73.6% for patients with positive fFN test results and 52.9% for patients with a cervical length < 25 mm (Table 2).

**Table 2:** Outcome comparison of positive test patients

	PAMG-1-positive *n* = 15	fFN-positive *n* = 19	Cervical length < 25 mm *n* = 17
Mean delivery interval (days)	6.8 ± 6.1	6.4 ± 5.9	17.7 ± 9.8
Delivery rate within 7 days	73.3%	73.6%	52.9%
Mean number of gestational weeks at diagnosis of threatened preterm birth	29.6 ± 2	30.4 ± 1.1	31.2 ± 0.4
Mean number of gestational weeks at delivery	30.1 ± 0.4	31 ± 0.3	32 ± 0.8

According to the primary outcome measure (delivery within 7 days), the sensitivities of PAMG-1, fFN and CLM were 73.3%, 73.6%, 52.9%, respectively, and the specificities of PAMG-1, fFN and CLM were 92.9%, 94.3%, and 90.9%, respectively. The PPVs of PAMG-1, fFN and CLM were 73.3%, 82.3%, 64.2%, respectively, and the NPVs of PAMG-1, fFN and CLM were 92.9%, 90.9%, and 86.2%, respectively (Table 2). PAMG-1 was not statistically superior to fFN with respect to SN, SP and NPV (*p* > 0.05) for its ability to predict delivery within 7 days (Table 3).

**Table 3:** Comparison of PAMG-1, fFN and cervical length < 25 mm in terms of delivery within seven days

Metrics (95% CI)	PAMG-1	fFN	Cervical length < 25 mm
SN	73.3%	73.6% ^a^	52.9% ^b c^
SP	92.9%	94.3%^a^	90.9% ^b c^
PPV	73.3%	82.3%	64.2% ^b c^
NPV	92.9%	90.9% ^a^	86.2% ^b c^
Validity of test	88.8%	88.8% ^a^	81.9% ^b c^

^a^ PAMG-1 vs fFN *p* > 0.05

^b^ PAMG-1 vs cervical length < 25 mm *p* < 0.05

^c^fFN vs cervical length < 25 mm *p* < 0.05

The test validity for PAMG-1, fFN and CLM regarding their prediction for delivery within 7 days was 88.8%, 88.8%, and 81.9%, respectively (Table 3).

The mean delivery intervals in PAMG-1-positive women, fFN-positive women and women with a cervical length < 25 mm were 6.8 ± 6.1, 6.4 ± 5.9 and 17.7 ± 9.8 days, respectively. No significant difference was detected between fFN and PAMG-1 in terms of the time-to-delivery interval (*p* > 0.05) (Table 1). No adverse events were exist from performing the tests.

## Discussion

Worldwide, approximately 15 million children are born prematurely each year (range 12 to 18 million) [9]. Of these preterm births, 84% occur at 32 to 36 weeks, 10% occur at 28 to <32 weeks, and 5% occur at <28 weeks of gestation. Due to recent technological and pharmacological advances in perinatal and neonatal care, the neonatal and perinatal mortality rates have dramatically decreased.

Although such advances have improved the life expectancy of premature babies, they have come at a high financial cost [10, 11]. These costs are much more problematic in developing countries such as Turkey. A study conducted in a tertiary care centre in Turkey reported that the mean total hospitalization cost and the daily cost of a preterm birth was $4187 and $303, respectively [12]. Some have attempted to reduce costs in developed countries using the Very High Human Development Index (VHHDI). In the United States, according to a committee for the Institute of Medicine, annual costs are estimated to be at least $26.2 billion. Consequently, the March of Dimes initiated the Prematurity Campaign in 2003, and in 2008, this organization established a goal for the reduction of the US PTB rate to 9.6% by 2020 [13].

The exact diagnosis and the treatment options can reduce preterm birth rates and costs. Recently, the measurement of cervical length and fFN tests have been accepted as useful for the support or exclusion of the diagnosis of preterm labour when the diagnosis is unclear in selected patients [14]. In this observational study on diagnostic test accuracy, we compared the performance of vaginal PAMG-1 protein detection with that of fFN and CLM in terms of delivery within 7 days. According to our results, the diagnostic accuracy of PAMG-1 regarding delivery within 7 days is similar to that of fFN.

In the literature, only two trials that investigated the presence of cervicovaginal PAMG-1 for the same purpose have been published. Although both have reported that PAMG-1 protein can be used as a predictor of preterm delivery, the SP, SN, PPV and NPV are different in each study [15–17]. In contrast to our results, both of those studies reported higher SN and SP values for PAMG-1 compared with fFN regarding their ability to predict delivery within 7 days. We believe that this is due to our strict patient selection criteria. As a comparison, the study by Nikolova et al. included self-reported signs or complaints of PTB without electrocardiotocographic confirmation [16]. Ehsanipoor et al. selected patients who were dilated up to 6 cm and in whom the fFN test should not be applied [17]. These authors also declared that these unrestricted patient selection criteria were limitations of their studies. In the present study, women with the following clinically evaluated symptoms of preterm labour were included: at least 4 contractions in 60 min based on external electrocardiotocography, cervical dilation of >1 cm to <3 cm, effacement of >50%, and a cervical length < 30 mm on transvaginal ultrasound. Similar cervical diameters were also selected as inclusion criteria in the study by Van Baaren et al. They revealed that measurement of cervical length combined with foetal fibronectin testing in cases of a cervical length between 15 and 30 mm, improves the identification of women with a low risk for spontaneous delivery within 7 days [18].

According to the results of the present study, the performance of PAMG-1 was lower than that of fFN in patients with short cervical length. This result is also different from those of other studies [16, 17]. We believe that this difference is also due to our inclusion criteria. A dilation of up to 6 cm is not a suitable cut-off for fFN accuracy. Our selection criteria were suitable for both fFN and PAMG-1 assessment. As a result, we believe that the comparison of the PAMG-1 and fFN test results is much more reliable in the present study.

The strengths of our study are its prospective nature and strict criteria, which allow for much more accurate comparisons between fFN and PAMG-1. The subjects in the present study had no history of preterm birth, which can also strengthen the accuracy of the results. Shorter cervical length measurements are common in women with a history of preterm birth. A limitation of our study was its small sample size. This study was time-bound, and this is why we stopped once 72 women were included. Although we stated this as a limitation, it is clear that our sample size is larger than those of the other two reported trials that compared both PAMG-1 and fFN [16, 17]. Since the patient selection criteria were unrestricted in the other studies, patients who were eligible for one test may not have been suitable for another test.

## Conclusions

As a conclusion, PAMG-1 detection in cervicovaginal secretions may predict preterm labour within 7 days. However, fFN detection is also successful in this regard. Future studies with larger sample sizes are warranted to confirm these results.

## Abbreviations

CI: Confidence interval
CLM: Transvaginal cervical length measurement
fFN: Foetal fibronectin
NPVs: Negative predictive values
OR: Odds ratio
PAMG-1: Placental alpha microglobulin-1 protein
PPVs: Positive predictive values
PTB: Preterm birth
SNs: Sensitivities
Sps: Specificities
